# Functional protein divergence in the evolution of *Homo sapiens*

**DOI:** 10.1186/gb-2008-9-2-r33

**Published:** 2008-02-15

**Authors:** Nuria Lopez-Bigas, Subhajyoti De, Sarah A Teichmann

**Affiliations:** 1 Research Unit on Biomedical Informatics, Experimental and Health Science Department, Universitat Pompeu Fabra, Dr. Aiguader, Barcelona, 08003, Spain; 2 MRC Laboratory of Molecular Biology, Hills Road, Cambridge CB2 2QH, UK

## Abstract

**Background:**

Protein-coding regions in a genome evolve by sequence divergence and gene gain and loss, altering the gene content of the organism. However, it is not well understood how this has given rise to the enormous diversity of metazoa present today.

**Results:**

To obtain a global view of human genomic evolution, we quantify the divergence of proteins by functional category at different evolutionary distances from human.

**Conclusion:**

This analysis highlights some general systems-level characteristics of human evolution: regulatory processes, such as signal transducers, transcription factors and receptors, have a high degree of plasticity, while core processes, such as metabolism, transport and protein synthesis, are largely conserved. Additionally, this study reveals a dynamic picture of selective forces at short, medium and long evolutionary timescales. Certain functional categories, such as 'development' and 'organogenesis', exhibit temporal patterns of sequence divergence in eukaryotes relative to human. This framework for a grammar of human evolution supports previously postulated theories of robustness and evolvability.

## Abstract 2

Quantification of the divergence of proteins by functional category shows that morphological changes in metazoa have been driven by variation in regulatory genes.

## Background

Though it is known that coding regions evolve primarily by sequence divergence of individual genes and gene gain and loss, altering the gene content of the organism, it is not well understood how these processes have resulted in the tremendous diversity of metazoa present today. Have metazoans evolved through a process of incremental changes occurring evenly across genes from different functional categories, or is there a pattern by which some classes of gene function accumulate mutations quickly, while others remain conserved throughout evolution across different branches of the species tree?

The differential rate of evolution of proteins has been of long-standing interest. As early as 1971, Dickerson [1] studied the relationship between the number of amino acid differences and divergence time for cytochrome c, hemoglobins, and fibrinopeptide. In 1978, Dayhoff *et al*. [2] studied the rates of amino acid substitutions per amino acid site in various proteins, finding that histones are among the most conserved proteins while hormones and immunoglobulins evolve the fastest.

The recent availability of full genome sequences has allowed large scale comparisons of gene sequences between organisms, that is, chicken and human [3], human and mouse [4] or human, fugu, *Drosophila *and *Caenorhabditis elegans *[5]. Purifying selection on mutations has been related to a number of aspects of protein structure and function, such as the number of interaction partners, expression levels, dispensability, and the character and interface of protein-protein complexes amongst others (reviewed in [6]). Recent studies have focused on sequence divergence between humans and other primates, and on the sequence diversity within human populations in the form of single nucleotide polymorphisms [7-13]. Another approach to quantifying evolutionary divergence at the genome level is to measure gene gains and losses between species by identifying orthologs between pairs of organisms [14]. Other studies have analyzed specific functional gene sets in particular groups of organisms. For example, Babu *et al. *[15] reported high variability in prokaryotic transcription factor repertoires in contrast to the conservation of their target genes in prokaryotes. Coulson and Ouzounis [16] reported that eukaryotic transcriptional regulator families are primarily taxon-specific. Peregrin-Alvarez *et al*. [17] found that metabolic enzymes are present across a wider phylogenetic spectrum than other genes.

Some studies have analyzed the expansion of protein families in relation to the number of genes in the genome [18,19] or to organismic complexity, measured as the number of cell types [20]. These studies report that protein families involved in regulatory processes and extracellular functions show an increase in the number of genes correlated with genome size and organismic complexity.

**Figure 1:**
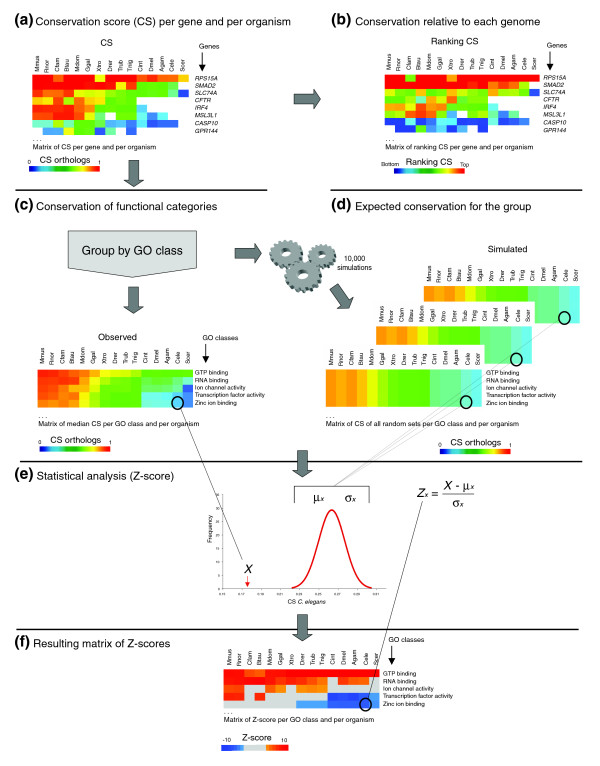
Flow chart of the FRED method for analyzing the protein divergence landscape of functional categories. **(a) **We start from a matrix of all human genes with the conservation score (CS) in each of the 15 genomes analyzed. **(b) **First, all genes with a CS over 0 are ranked in each organism, and the highly ranked genes are shown in red and lowly ranked in blue following a gradient of colors. White cells mean that no ortholog/homolog is detected. Next, the genes are classified according to GO terms. **(c) **For each set of genes within a GO category, we calculate the median CS, and also select 10,000 sets of the same number of genes as in the GO category considered at random from the complete set of genes with GO annotation. **(d) **For each random set, we calculate the median CS. **(e) **From the 10,000 random sets we obtain the expected median CS and the standard error, which allow us to calculate the Z-score for the GO category under consideration. **(f) **This Z-score is then plotted in a matrix on a color-coded scale. Gray means no significant difference in the level of conservation compared to the background. A similar procedure is followed for the calculation of Z-scores for number of orthologs and homologs by counting the proportion of genes with homologs or orthologs in each set. Mmus, *Mus musculus*; Rnor, *Rattus norvegicus*; Cfam, *Canis familiaris*; Bta, *Bos taurus*; Mdom, *Monodelphis domestica*; Ggal, *Gallus gallus*; Xtro, *Xenopus tropicalis*; Drer, *Danio rerio*; Trub, *Takifugu rubripes*; Tnig, *Tetraodon nigroviridis*; Cint, *Ciona intestinalis*; Agam, *Anopheles gambiae*; Dmel, *Drosophila melanogaster*; Cele, *Caenorhabditis elegans*; Scer, *Saccharomyces cerevisiae*. All the results of these analyses for all GO categories are provided online in a searchable database at [28].

All of the studies mentioned above address the evolution of the protein repertoire in various sets of organisms. Here, we investigate the evolutionary divergence of all functional groups of human proteins at different levels. We measure divergence of human proteins relative to 15 eukaryotes spanning from mammals to fungi using a new method we call FRED (for 'Functional categories and their relative evolutionary divergence'), which is outlined in Figure 1.

In contrast to previous studies of conservation [7-9,13,14,21], our work spans 15 genomes ranging from mammals to fungi, which allows us to gain a broad perspective on the history of mutations and selection leading to the human lineage. By comparing the sequence evolution of different functional groups in human we see which parts of the genome are more plastic to change, and which parts are conserved. With our approach, we gain a dynamic picture of the extent of conservation, since some functions are strictly conserved only within vertebrates, for instance, while others are maintained across all eukaryotes. This dynamic picture of differential rates of divergence across functional categories reveals a 'grammar' of metazoan evolution up to the human lineage.

## Results

### Functional protein divergence in the glucagon and insulin signaling pathways

First, we focus on a specific system to investigate whether there are different levels of protein conservation for types of protein function. Glucose metabolism is a key process in human cells for the storage and release of energy. The enzymes that catalyze the reactions leading to the synthesis and degradation of glucose are regulated by complex signaling pathways triggered by glucagon and insulin hormones [22]. This system is a good case study, as there are a variety of proteins of different molecular functions involved in the process: enzymes, receptors, signal transducers and transcription factors.

**Figure 2:**
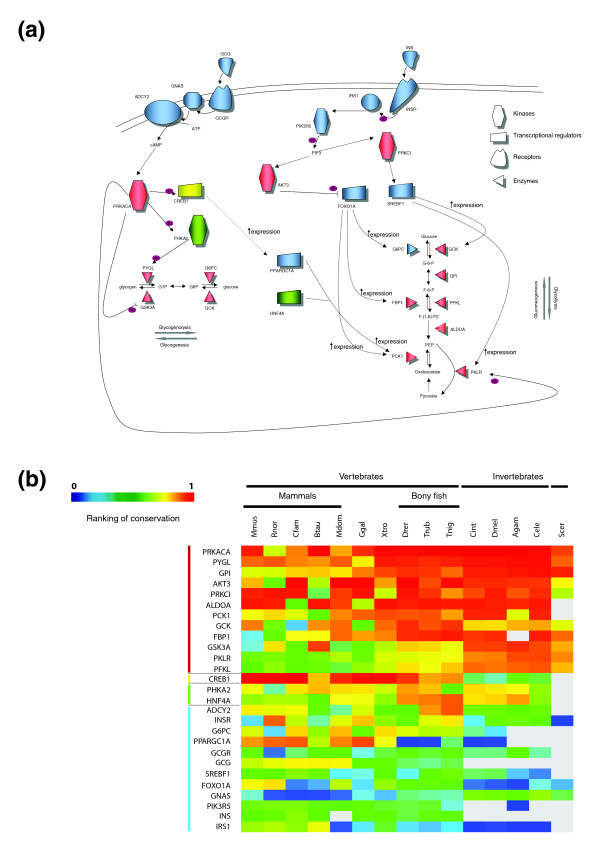
Degree of conservation of the glucagon and insulin signaling pathways. **(a) **Regulatory interactions between proteins involved in glucagon (GCG) and insulin (INS) signaling, and enzymes involved in glucose and glycogen metabolism. Proteins depicted in red show high conservation, those depicted blue have low levels of conservation and the ones in green intermediate conservation. The CREB protein is represented in yellow because it is highly conserved in vertebrates and not in invertebrates. There is a clear correlation between the functions of the molecules shown in the key and the degree of conservation indicated by the color code: enzymes and kinases tend to be red and conserved, while signal transducers, receptors and transcription factors tend to be blue and divergent. **(b) **Matrix of normalized ranking of the genes depicted in (a). The rows in the matrix are ordered by the sum of the CS rank in the 15 organisms.

Figure 2a depicts key genes involved in glucose homeostasis regulated by insulin and glucagon hormones and the functional relationships between them. We measure sequence divergence using the ranked 'conservation score' (CS; please refer to Materials and methods for a definition of this score; Figure 2b). This case study illustrates that the proteins involved in regulation of the glucose metabolism, such as glucagon (GCG) and insulin (INS), their receptors (INSR and GCGR) and some transcriptional regulators involved in the process (SREBF1, PPARGC1A and FOXO1A), have very low conservation compared to enzymes directly involved in the catalysis of reactions of glucose metabolism. For example, FOXO1A is a transcription factor that regulates the expression levels of several enzymes in gluconeogenesis (G6PC, FBP1 and PCK1). This protein is highly divergent in eukaryotes, especially in invertebrates. Recently, this gene has been shown to be differentially expressed between primates [23].

The mammalian insulin signaling pathway has been studied in depth. Insulin signaling influences other processes in addition to glucose metabolism, such as protein synthesis, growth and cell division. This pathway, from the receptor to the target serine/threonine kinases, is known to be conserved in fruitfly [24] and nematode [25-27]. However, as seen in our analysis, the degree of conservation of the proteins involved in the insulin pathway is low relative to the conservation of other human proteins. In contrast, most of the enzymes involved in the process are highly conserved across eukarya.

Thus, the human proteins involved in this well-known biological system show the following trend of conservation across metazoa: genes whose function is the direct catalysis of enzymatic reactions are conserved, while genes involved in the regulation of these core catalytic proteins (receptors, signal transducers and transcription factors) are divergent. We wondered whether this pattern of conservation is specific to the insulin and glucagon signaling system, or whether it is a general pattern found in the whole human proteome.

### Protein divergence of the human proteome during mammalian evolution

**Figure 3:**
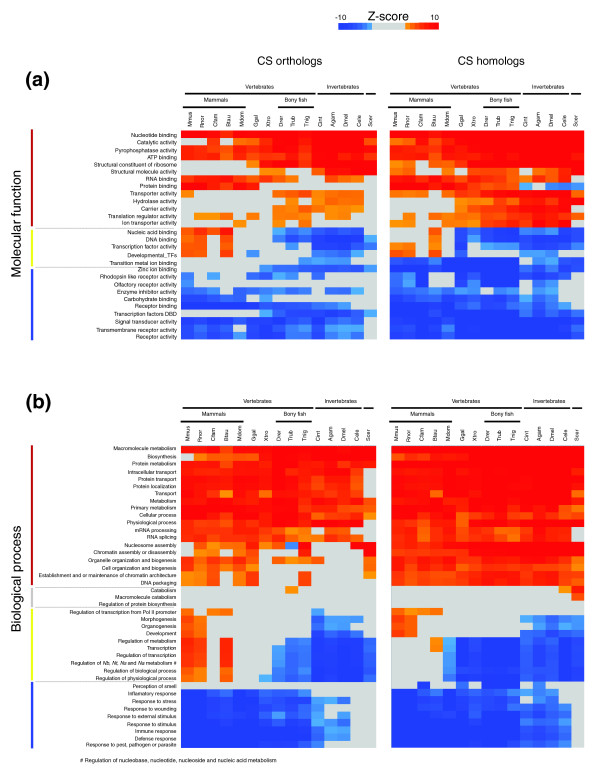
Divergence of orthologs and homologs of representative functional categories. **(a) **Molecular function and **(b) **biological process. Colors towards red signify high relative conservation of the group of genes in a particular genome. Colors towards blue signify low relative conservation. Gray means no statistically significant difference in conservation level compared to the background of the rest of the genome. White cells denote that there is no gene with the GO term and with ortholog/homolog in the other organism. The colored lines on the left of the names of the functional classes correspond to the colors of the categories represented in Figure 5.

To generalize our study of evolutionary rates across functional categories, we analyzed the evolutionary landscape of human genes and their orthologs in over 300 functional categories across 15 eukaryotes using our FRED method. We quantified the conservation of genes using four measures. Two of the measures are the CS of the orthologs (Figure 3, and Figures S2a,b and S3a,b in Additional data file 1) and the CS of the closest homolog (Figure 3, and Figures S2d,e and S3d,e in Additional data file 1) of human proteins in other eukaryotic organisms. These two measures of amino acid sequence conservation are highly correlated across all genomes compared to human (for example, the correlation coefficient = 0.97 for mouse and 0.93 for *Drosophila*; Table S1 in Additional data file 1). In addition to these measures of sequence divergence, we quantified the presence of orthologs to the human proteins in the other genomes as well as the presence of homologs. The degree of orthology reflects the presence of human genes in other genomes (Figures S1, S2c and S3c in Additional data file 1), while homology reflects the presence of the human gene family in other genomes (Figures S1, S2f and S3f in Additional data file 1) (Please refer to the Supplementary methods in Additional data file 1 for details of the calculation of these measures.). The relative conservation of Gene Ontology (GO) categories, the statistics and the CS for each of the 14,062 human genes analyzed are provided online in a searchable database [28].

**Figure 4:**
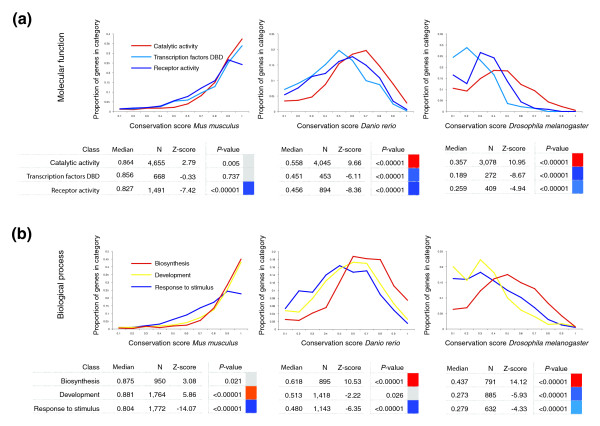
Histogram distribution of CSs of orthologs for selected GO categories in *M. musculus*, *D. rerio *and *D. melanogaster*. **(a) **The CS distributions for proteins in three molecular function categories. 'Catalytic activity' is significantly conserved in all three organisms, while 'Transcription factors DBD' and 'Receptor activity' are significantly divergent in zebrafish and *Drosophila*. **(b) **The CS distributions for proteins in three biological process categories. 'Biosynthesis' is a highly conserved category in all three organisms, while 'Development' is significantly conserved in mouse but significantly divergent in *Drosophila*. 'Response to stimulus' is significantly divergent across all three organisms.

While we also consider the rate of loss of genes and families, we focus on sequence divergence in describing our results. Though there is a wide range of protein sequence conservation in all categories, the shape of the distribution differs between categories (Figure 4). Some categories contain many highly conserved proteins across all eukaryotes and only few proteins that are not conserved, while others are dominated by proteins that diverge quickly. Yet another type of category contains proteins that are conserved in one phylogenetic group relative to human and divergent in another phylogenetic group.

The set of proteins in a particular functional category will vary when comparing human to different genomes due to gene gain and loss. Therefore, the average CS of the proteins in a functional category depends on both the sequence divergence of ancient conserved orthologs, as well as genes that have evolved anew in the lineage considered (and are still conserved in human). Since this is true of all functional categories, the median conservation score of a category is a robust measure of variation in proteins between the functional categories of a particular pair of genomes. In order to make sure that our conclusions hold even when we consider only universally conserved orthologs, we have used the KOGs classification [29] as a further control data set (Figure S4 in Additional data file 1). We will now survey the varying patterns of conservation of the different functional categories, and will attempt to identify underlying principles.

### Metabolism, transport and cell structure proteins are conserved

The most conserved functional categories, shown in red in Figure 3, belong to the three super-groups of 'metabolism' (catalytic activity, macromolecule metabolism, biosynthesis, protein metabolism, primary metabolism, mRNA splicing, mRNA processing), 'transport' (transporter activity, carrier activity, ion transport activity, protein transport, intracellular transport, transport, protein localization) and 'cell structure' (structural constituent of ribosome, structural molecule activity, nucleosome assembly, organelle organization and biogenesis, chromatin assembly or disassembly, DNA packaging) (Figure 3). Note that the nucleosome assembly category is actually more conserved than indicated by CS orthologs in Figure 3 (see CS homologs in Figure 3); this distortion is due to a known limitation of the current Ensembl-Compara ortholog assignment (Albert Vilella, personal communication). Many of the proteins that belong to conserved categories involved in binding are also part of the metabolism super-group: nucleotide binding, ATP binding, RNA binding, protein binding. The three broad groups of metabolism, transport and cell structure are all core cellular processes.

### Modulators of core processes are highly divergent

In contrast, proteins with the lowest conservation, shown in blue in Figure 3, are mainly involved in 'regulatory' functions and processes or involved in 'responses to the environment'. For example, amongst the least conserved molecular functions are receptors (receptor activity, transmembrane receptor activity, olfactory receptor activity, rhodopsin like receptor activity), signal transducers and transcription factors not involved in development (TF-DBD). All these divergent molecular functions are mainly involved in modulating the conserved core processes. Note that this trend holds across many different structural families and different types of regulators, so that it cannot be explained in terms of particular molecular characteristics, such as fewer constraints on the fold or on the surface of receptors compared to the constraints on enzyme.

The other biological processes that are evolving rapidly are those involved in responses to the environment: defense response, immune response, response to stimulus, response to external stimulus, response to stress, response to wounding, inflammatory response. These processes are all involved in the response to an external challenge to the organism. Since challenges such as parasites vary from one eukaryote to another, natural selection favors rapid evolution of proteins in these categories. Pressure for adaptation against changing environments and pathogens imposes strong selection for advantageous mutations to sweep the population rapidly.

Host-parasite interactions have been suggested as the most likely reason for the fast evolution of human immunity genes [21]. In addition, these categories have a low number of genes with orthologs or homologs in other organisms (Figure S1 in Additional data file 1). This is not surprising since the antibody-based immune system does not exist in bony fish or other lower eukaryotes [30], and there may be parallel or alternative adaptive immune systems in these organisms, such as the leucine-rich repeat receptor system in lampreys [31].

### Developmental genes are conserved only in mammals

Some categories are conserved amongst mammals while diverging rapidly in other organisms relative to human, including development, morphogenesis, organogenesis, and many categories associated with transcriptional regulation (Figures 3 and 4). Although the GO category 'transcription factor activity' shows this pattern of conservation, our division of transcription factors into 'Developmental TFs' and 'TF-DBD', which are transcription factors involved in other processes (see Materials and methods for details), reveals a fundamental difference between the two groups: transcription factors involved in development are highly conserved in mammals, while those not involved in development have only average conservation in mammals and are significantly divergent in bony fish relative to human. Both groups of transcription factors ('Developmental TFs' and 'TF-DBD') are highly divergent in invertebrates relative to human (Figure 3). Thus, for the transcription factors not involved in development, the trend is consistent with that of other regulatory categories such as receptors and signal transducers.

An example of a particular developmental transcription factor that is highly conserved in mammals but switches to being very divergent in invertebrates is the Neurogenic differentiation factor 1 (NeuroD1), which is required for dendrite morphogenesis and maintenance in the cerebellar cortex [32]. Note that there is a distribution of sequence conservation within each functional category. Thus, within the 'development' category, there are proteins, including some transcription factors, that form part of the core of the developmental network, conserved from invertebrates to human, while at the same time, other parts of the network have undergone profound change or innovation [33]. This trend is also noticeable when we focus on short evolutionary distance from humans. Though NeuroD1 follows the same pattern as the 'Developmental TF' category as a whole, there are exceptions: some transcription factors involved in core developmental processes, such as Pax-6, are remarkably conserved across all eukaryotes (Figure S2 in Additional data file 1).

### Controls for orthology, conservation measure, functional classifications and expression

This whole genome divergence pattern is not an artifact of the set of orthologs and homologs or the functional classification scheme we use: almost identical results are obtained using the orthologous groups within seven eukaryotes and also the functional classification provided by the KOGs [29] (Figure S4 and Table S1 in Additional data file 1). Furthermore, when we restrict the orthologous groups considered to those that are present across all seven eukaryotes, very similar results are obtained (Figure S4c,d in Additional data file 1). This means that orthologs present, for example, in mammals only are not distorting the general trend of a conserved core and divergent regulatory functions. In addition, this test also accounts for possible genome annotation biases due to conservation.

We compared our results of protein divergence (using CS) with two measures of divergence at the DNA level: the GERP (Genome evolutionary rate profiling) method developed by Cooper and colleagues [34] where divergence rate of every base position is compared against an expected rate; and the rate of non-synonymous substitution (dN) between human and mouse and human and rat genomes (see Materials and methods for details). Using the GERP score we measured divergence in mammals (chimpanzee, macaque, rat, mouse, dog, cow, opossum) at each coding base for around 15,000 human genes. An evolutionarily conserved base position has a low GERP score while a divergent position has a high score. We measured the average GERP score for all coding nucleotides for each gene and assessed whether these values differ significantly between functional groups using Z-score analysis (Figure S5 in Additional data file 1). Similarly, we assessed the dN values per gene. The FRED results using the GERP score and dN correlates remarkably well with that of the CS in mammals, showing consistency between DNA and protein-level measures.

Another factor that could potentially confound our analysis is the correlation between phylogenetic extent and expression breadth across tissues [35]. Restricting our FRED analysis to housekeeping genes (defined as genes expressed in more than 75 tissues in GeneAtlas v2 expression array [36]) or to genes with tissue-specific expression (those expressed in less than 20 tissues) yields essentially the same results as considering all proteins (data not shown).

From these control analyses, it is clear that our general conclusion is independent of factors such as expression, the definition of orthologs, the measure of conservation and the functional classification scheme used.

## Discussion

**Figure 5:**
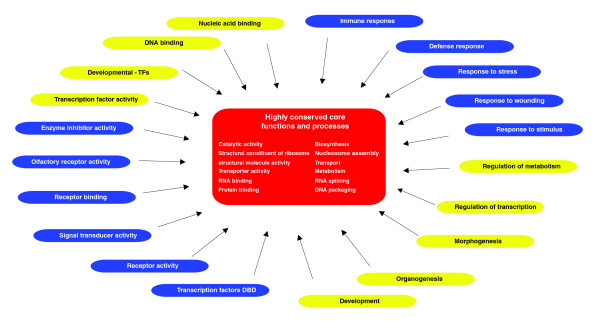
Peripheral and core functional categories. A set of core molecular functions and biological processes that are highly conserved are represented in red in the centre of the figure. Other sets of functions and processes that are highly divergent across all eukaryotes (blue) or highly divergent in some organisms and highly conserved in others (yellow) are represented on the periphery as regulators of the core processes. The colors correspond to the colored lines on the left in Figure 3.

Here, we establish the entire divergence landscape of human protein-coding genes across eukaryotes and discuss evolutionary selection in the human lineage in the light of long-term evolution. The main results from our analysis are, first, that two main groups of proteins are diverging at different speeds: regulatory proteins diverge quickly across species, while proteins involved in core processes are conserved (Figure 5). This shows that at the level of protein-coding genes, morphological changes in metazoan evolution relative to humans have been primarily driven by variation in regulatory genes rather than enzymatic and structural genes. While this is consistent with many previous small-scale studies, our conclusions are more comprehensive and clear due to the amount of data we consider in this analysis. Second, certain functional categories exhibit dynamic patterns of sequence divergence across their evolutionary history leading to human. Genes involved in 'development' and 'organogenesis' are significantly conserved within mammals, while significantly divergent in human relative to invertebrates.

The picture of long-term evolution is broadly consistent with the signatures of selection on functional categories in the human lineage, implying that the same underlying grammar guides the dynamics of metazoan evolution relative to human over short as well as long evolutionary timescales. Recent studies [7-10,13,21] have identified several categories of genes with higher divergence rates between hominids, or with signs of positive selection using different measures for detecting selection. For example, Bustamante and colleagues [7] identified genes involved in transport and cell structure as being under negative selection in the human lineage, which we find to have strong protein sequence conservation. Genes involved in regulatory processes have been reported to show signatures of positive selection [7,12], especially genes related to regulation of transcription. In addition, genes involved in immune and defense response also show strong signatures of positive selection in the human [7,12,21]. Both sets of categories have high sequence divergence in our comparison of human proteins to other eukaryotes. Bustamante *et al*. [7] report strong negative selection on ectoderm development, and we find development to be conserved in mammals.

Our analysis highlights some general systems-level characteristics of metazoan evolution relative to human: regulatory processes, such as signal transducers, transcription factors and receptors, have a high degree of plasticity, while core processes, such as metabolism, transport and protein synthesis, are largely conserved. This dual architecture of a conserved core and variable regulatory peripheral processes may confer robustness [37] and evolvability [38] on the system. So far there has been little concrete biological data on genes that are responsible for phenotypic plasticity and the evolution of species. Our analysis provides evidence for which genes and functional categories are most variable in organismic evolution, and what we observe fits the framework of the theories cited above.

We have focused purely on molecular variation of protein coding genes. There are many other dimensions to human evolution that are not captured by this analysis, or at least not directly. These include evolution of epigenetic regulation, alternative splicing, non-coding genes such as microRNAs [39,40], promoters [41,42] and other non-coding regions [43,44]. However, divergence rates of the human protein-coding genes are linked with evolution of all of these other processes, and proteins themselves are the major players in the development, structure and physiological adaptability of animals.

In the future, analyses such as ours will be aided by greater density of sequence information. The 'thousand dollar genome' or, more generally, cheaper sequencing and genotyping technologies will provide us with greater coverage of polymorphisms both in human and other organisms. At the same time, population-wide measurements of expression at the RNA [45-47] and protein levels will allow us to understand how changes in DNA affect cellular processes. In terms of whole organisms, quantitative phenotypic traits are beginning to be related to sequence features [48]. Ultimately, we hope to understand what variations at cellular, organismic and species level are determined by genomic diversity.

## Materials and methods

### Functional classification

Protein-coding human genes with functional annotation (14,062 genes) were extracted from Ensembl [49] for both the 'molecular function' and 'biological process' GO classification [50]. Note that GO terms are organized into structures called directed acyclic graphs, such that a specialized term can be associated with several less specialized terms. For instance, a gene annotated with the term 'transcription factor activity' will also automatically be annotated as 'DNA binding' and 'nucleic acid binding', which are parent terms of the former terms in the GO database. We analyzed all the GO molecular function categories and all biological process categories that have more than 100 human genes annotated with their term (135 and 242, respectively). We carried out our analysis with and without annotation that is inferred electronically (this represents 54% of annotations for biological process and 69% for molecular function), and the conclusions are consistent using both versions of GO annotation (data not shown). In addition, although we used all the Ensembl genes with GO annotations (14,062 genes) for our study, we repeated the analysis using only genes included in the RefSeq database [51], finding essentially the same results (data not shown).

In addition to these categories, we introduced a category of our own of predicted sequence-specific DNA-binding transcription factors. This category is defined from the database DBD, which contains repertoires of predicted transcription factors for completely sequenced genomes based on domain assignments from the SUPERFAMILY and PFAM hidden Markov model libraries [52]. Furthermore, we divided this category into: developmental transcription factors (that is, also annotated with the GO biological process term 'development'); and all others (TF-DBD).

In the alternative functional classification scheme considered, the KOGs database [29], there are orthologous groups of proteins from seven eukaryotic genomes: three animals (the nematode *C. elegans*, the fruit fly *Drosophila melanogaster *and *Homo sapiens*), one plant, *Arabidopsis thaliana*, two fungi (*Saccharomyces cerevisiae *and *Schizosaccharomyces pombe*), and the intracellular microsporidian parasite *Encephalitozoon cuniculi *[29]. The orthologous groups of proteins are classified into one of 25 functional categories wherever possible, so there are fewer larger categories in this scheme compared to the GO scheme.

### Genomes and phylogenetic groups

Proteins from the human genome (NCBI36 - Ensembl v.42) were used for the analysis. In addition, the following versions of other completed eukaryotic genomes are part of our analysis. Mammals: two rodents, mouse (*Mus musculus *- NCBIM36) and rat (*Rattus norvegicus *- RGSC3.4), dog (*Canis familiaris *- CanFam2.0), cow (*Bos taurus *- Btau2.0), opossum (*Monodelphis domestica *- MonDom4.0). Other vertebrates: chicken (*Gallus gallus *- WASHUC2) and Frog (*Xenopus tropicalis *- JGI4.1), and three bony fish, zebrafish (*Danio rerio *- Zv6), fugu (*Takifugu rubripes *- FUGU4) and tetraodon (*Tetraodon nigroviridis *- TETRAODON7). Invertebrates: mosquito (*Anopheles gambiae *- AgamP3), fruitfly (*D. melanogaster *- BDGP4.3), sea urchin (*Ciona intestinalis *- JGI2), worm (*Caenorhabditis elegans *- WS160) and yeast (*S. cerevisiae *- SGD1.01).

### Homology and orthology

Pairwise similarity for all human genes against each other and the other genomes was obtained from Ensembl-Compara (v42.0) [49]. These relationships were computed using WUBLASTP (v2.0) [53] for each gene considering the longest peptide isoform only. Homologs are defined as those sequences with a match with an E-value ≤10^-10 ^in other genomes, while orthologs in Ensembl-Compara are assigned based on maximum likelihood phylogenetic gene trees.

The presence of an ortholog for a human protein in another genome provides more precise information about the conservation of the protein than the presence of a homolog. However, orthology detection is error-prone for distant evolutionary relationships and for protein families with many duplications and losses. Therefore, we decided to use both, orthologs and homologs, in our study.

### Functional categories and their relative evolutionary divergence: FRED

We have developed a scheme for quantifying the relative protein sequence divergence of different functional categories between a pair of genomes, which we used to compare human to 15 other genomes. We call this framework FRED, for functional categories and their relative evolutionary divergence, which is outlined in Figure 1 and described below.

#### Conservation measures

The primary measure of evolutionary rate that we use is amino acid sequence evolution through the CS [54]. We use the median CS for all the orthologs of human to another genome in a particular functional category. This means that the set of proteins considered for a particular functional category can differ when comparing human to one genome or another due to gain and loss of genes throughout evolution. However, we normalize the rate of sequence divergence for each functional category by the average divergence for the pair of genomes considered. This means that gene gain and loss in a particular category is implicitly compared to the rates in other functional categories when we study sequence divergence. Furthermore, we also consider the set of universally conserved orthologs across eukaryotes from the KOGs database [29] and test our conclusions on this set. In addition, we have analyzed the extent of the existence of orthologs and homologs to the human genes in the 15 other genomes, and discuss the extent of correlation between the different measures of evolutionary rate.

#### Conservation score

The CS is an estimate of the divergence that has occurred between a pair of proteins during evolution, and is independent of the length of the proteins [54]. The value of CS was calculated for each human gene by dividing the WUBLASTP score of the ortholog (or the closest homolog) in the other organism by the WUBLASTP score of the protein against itself, as reported elsewhere [54]: CS ortholog or homolog = WUBLASTP score ortholog or homolog/WUBLASTP score self.

The CS accounts for the proportion of the query protein matched by WUBLASTP and the quality of the match, but is independent of the query protein length. The CS ranges from 0, when no ortholog or homolog is detected, to 1, when the closest homolog is identical to the human protein. Note, that for all our analyses using CS, we use only values higher than 0, meaning that we take into account only genes with detectable and significant orthologs or homologs. This score is indicative of how conserved a protein has remained through evolution, and hence the degree to which mutations within the sequence have been tolerated. We do not consider the molecular details of the differences in mutation rates, such as variations in the proportion of residues that are required for adequate protein function. Instead, we consider the net result of accepted mutations across functional categories.

In Figure 2b, we display in color the relative CS of each gene in a particular organism. To do this we ranked all human genes with homologs in the other genome according to their CS. The gene with the highest CS is shown in red and the one with the lowest CS in blue, with all others in intermediate colors according to their rank by CS. Thus, colors towards red mean high relative CS of the protein, green is medium relative CS and blue low relative CS.

Note that for the orthologs from the KOGs classification scheme, the CS value was calculated for each human gene by dividing the BLASTP score of the closest KOG partner in the other organism by the BLASTP score of the protein against itself.

#### Simulations for Z-score calculations

As described above and summarized in Figure 1, we grouped the genes by GO molecular function and biological process category, and then calculated both the mean and median CS for orthologs and homologs, as well as the number of genes with homologs or orthologs in a particular genome. To test whether there was a significant deviation from random expectation for these measures we used the Z-score:

$$Z_{x}=(X-\mu_{x})/\sigma_{\bar{x}}$$

where *μ*_*x *_is the mean, and $\sigma_{\bar{x}}$ is the standard error. The $\sigma_{\bar{x}}$ for number of genes with homologs and orthologs was calculated as:

$$\sigma_{\bar{x}}=\sqrt{\frac{\rho (1-\rho )}{N}}$$

where *ρ *is the proportion of genes in the category in question that have homologs or orthologs, and *N *is the total number of genes in the category. To calculate $\sigma_{\bar{x}}$ for the mean and median CS in each of the GO categories (*X*), we randomly selected 10,000 datasets of human proteins of identical sample sizes as the category in question and repeated the calculation for each random set. Z-scores for both mean and median CS values for functional categories yield essentially the same results, as the correlation coefficients between the two measures are greater than 0.9 for all functional categories and genomes.

We display matrices of Z-score values in which each cell is represented by a color-coded scale. Red signifies conservation (either greater number of homologs or orthologs than the background, or greater average conservation score than the background) and blue signifies divergence. Gray means no significant difference in the level of conservation compared to the background.

When adjusting the conventional *α* value (0.05, the *p*-value threshold) using the Bonferroni correction for multiple testing we obtain a corrected *α *of 1.3 × 10^-4^, taking into account that we are doing 377 tests (135 molecular function and 242 GO biological process categories). Therefore, we consider as significant absolute Z-scores larger than 3.652 (|Z| > 3.652), which corresponds to an analytical *p*-value of 1.3 × 10^-4^. This is a stringent threshold as Bonferroni is a conservative correction, especially for the data structure considered here.

Note that our measure of degree of conservation of a functional class (Z-score) is always relative to the conservation of all the genes in that genome in comparison to human. For instance, transcription factors are diverging rapidly in fly relative to human but have average conservation in mouse; this means that the orthologous fly-human transcription factors have diverged rapidly compared to fly-human orthologs in other functional categories, not compared to the mouse-human orthologs. The speed of divergence of a category will depend on the divergence of both ancient conserved genes and genes that have arisen within the particular lineage considered. Clearly, there will be more human orthologs that have arisen recently in organisms closely related to human. The expectation is that a recently duplicated gene will have a relatively high rate of sequence divergence in order to sub- or neo-functionalize. The contribution of such genes to various functional categories will be uneven, since it is known that some categories expand more quickly than others [18]. At the same time, the constraint on proteins in most functional categories will be more similar in organisms closer to human, and may change in organisms more distantly related to human (even if there are orthologs within the category). To control for these issues, we re-calculated the Z-scores for KOGs functional categories on proteins that are universally conserved across all seven eukaryotes in the KOGs database (see main text).

### Divergence profile of orthologous regions across mammals: GERP

To understand the evolutionary history of coding regions at base level positions, we considered the divergence profile of orthologous regions across mammals. We used the GERP method developed by Cooper and colleagues [34] where divergence rate of every base position is compared against an expected rate. An evolutionarily conserved base position has a low GERP score while a divergent position has a high score.

First, ortholog information for human genes in seven other mammalian genomes, namely chimpanzee, macaque, rat, mouse, dog, cow, opossum, was collected from Ensembl-Compara v37 and 42. We considered only orthologs that were >100 amino acids long aligned over at least 70% of the human protein. DNA level multiple alignment was performed using DIALIGN [55]. The neutral phylogenetic tree of the mammalian genomes was constructed by eliminating nodes that were not present in our study from the tree provided by Cooper *et al*. [34]. The average neutral rate of substitution for the mammalian genomes included in the analysis was taken as 1.93 substitutions per base. Semphy [56] was used by GERP to calculate the observed rate of divergence on a base-by-base basis. The score for evolutionary divergence was calculated as GERP score = Observed rate - Expected rate. Around 15,000 genes that had orthologs in 4 or more mammalian genomes were subject to GERP analysis.

We measured the average GERP score for all coding nucleotides for each functional category and we assessed using FRED analysis if these values differ significantly between functional groups (Figure S5 in Additional data file 1).

### Non-synonymous substitution rates: dN

dN data for the genes were taken from Ensembl-Compara v45 [49]. We computed the correlation between dN and CS for orthologs of human genes in mouse (16,040 genes) and rat (14,726 genes) (Figure S6 in Additional data file 1). We assessed using FRED analysis if the dN values for human-mouse and human-rat differ significantly between functional groups (Figure S5 in Additional data file 1).

## Abbreviations

CS, conservation score; FRED, Functional categories and their relative evolutionary divergence; GERP, Genome evolutionary rate profiling; GO, Gene Ontology.

## Authors' contributions

NLB calculated CSs for all human proteins and carried out Z-score analysis. SD performed the GERP analysis, compared CSs against other methods and helped to draft the manuscript. NLB and SAT conceived the study, participated in its design and coordination, and drafted the manuscript. All authors read and approved the final manuscript.

## Additional data files

The following additional data files are available. Additional data file 1 contains Supplementary Table 1, Supplementary Figures 1, 5 and 6, figure legends for all supplementary figures and screenshots of the evolvability web server [28], which contains the web-based figures, statistics and information on the CS for all genes.

## Supplementary Material

Additional data file 1 Supplementary table and figures. Click here for file
