# Supplementary material for: Functional protein divergence in the evolution of Homo sapiens
Source: Genome Biol. 2008 Feb 15;9(2):R33. doi: 10.1186/gb-2008-9-2-r33 (PMC2374701; doi:10.1186/gb-2008-9-2-r33)
Supplement: Additional data file 1 — Supplementary table and figures. [file gb-2008-9-2-r33-S1.pdf]

Supplementary Table 1. Correlation coefficients between different conservation measures. The Z-scores for each functional category in each organism are compared across the different measures of conservation described in the manuscript.

Supplementary Figure 1. Matrix of Z-scores for proportion of genes with orthologs and homologs of representative functional categories. (A) Molecular Function and (B) Biological Process. Red represents high relative proportion of genes with homologs or orthologs for the category in a particular genome and blue represents low relative proportion. Gray means no statistically significant difference compared to the background of the whole genome. White cells denote that there is no ortholog/homolog with the GO term in the other organism.

Supplementary Figure 2. Matrices of Z-scores for six conservation measures for all GO Molecular Function categories analysed. (A) Median CS for orthologs, (B) average CS for orthologs, (C) proportion of genes with orthologs, (D) median CS for homologs, (E) average CS for homolog and (F) proportion of genes with homologs. The colour code is as in Figure 4 and Supplementary Figure 1. This figure is based on HTML pages on the supplementary website (http://bg.upf.edu/evolvability). The link in the name of the GO category points to a page with details on the statistics of this category. In the statistics page, the link [Genes] points to a page with a coloured matrix of all genes in this category where the colour represents the rank CS of the gene in each genome. The link on the name of each gene will lead you to a page with the actual values of CS orthologs and CS homologs for that gene.

Supplementary Figure 3. Matrices of Z-scores for six conservation measures for all GO Biological process categories analysed. The legend for parts (A) to (F) are as for Supplementary Figure 2 above.

Supplementary Figure 4. Matrices of Z-scores for conservation measures using the KOG database. (A) Median CS for KOG orthologs for GO Molecular Function, (B) Median CS for KOG orthologs for GO Biological Process, (C) Median CS for universal KOG orthologs for GO Molecular Function, (D) Median CS for universal KOG orthologs for GO Biological Process and (E) Median CS for KOG orthologs for KOG functional annotation. The colour code is as in Figure 4 and Supplementary Figure 1. This figure is based on HTML pages on the supplementary website

(http://bg.upf.edu/evolvability). The link in the name of the KOG category points to a page with details on the statistics of this category.

Supplementary Figure 5. Divergence of orthologs of representative functional categories using three different divergence measures, CS orthologs, GERP and dN. (A) Molecular Function and (B) Biological Process. Colours towards red signify high relative conservation of the group of genes in a particular genome. Colours towards blue signify low relative conservation. Gray means no statistically significant difference in conservation level compared to the background of the rest of the genome. White cells denote that there is no gene with the GO term and with ortholog/homolog in the other organism. The coloured lines on the left of the names of the functional classes correspond to the colours of the categories represented in Figure 5.

Supplementary Figure 6. Correlation between dN and CS for orthologs of human genes in mouse (16040 genes) and rat (14726 genes). Data points are shown as diffused blue dots where intensity of blue colour reflects the density of data points in a particular region of the graph. Correlation co-efficient between dN and CS are shown at top left corner of respective graph.

Supplementary Figure 1

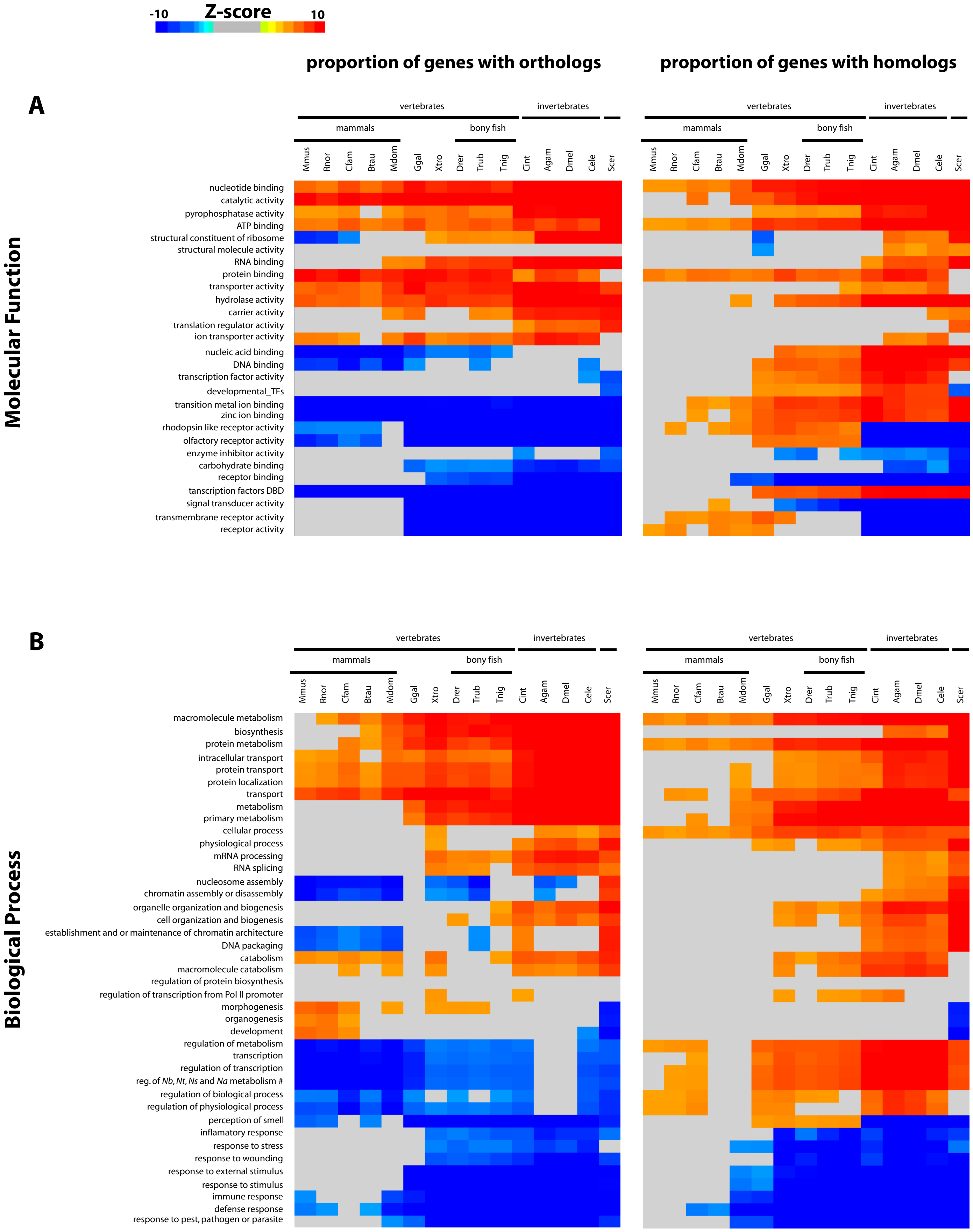

regulation of nucleobase, nucleoside, nucleotide and nucleic acid metabolism

Supplementary figure 5

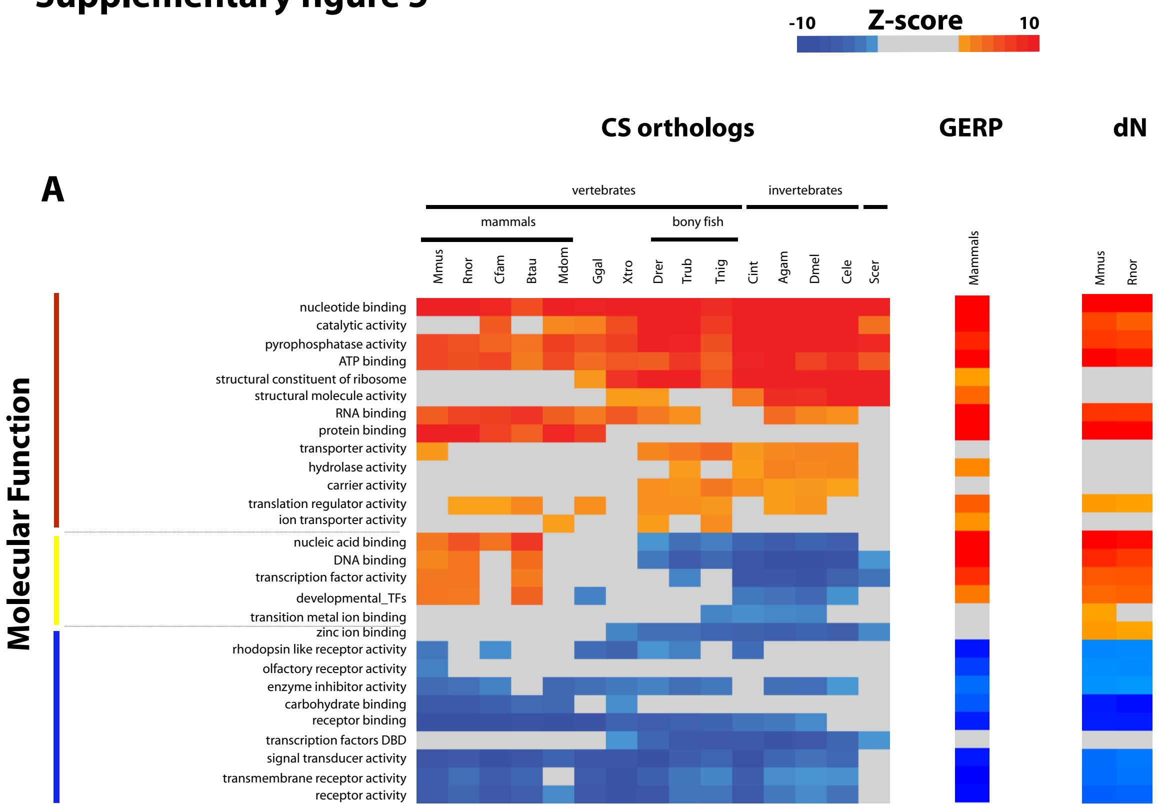

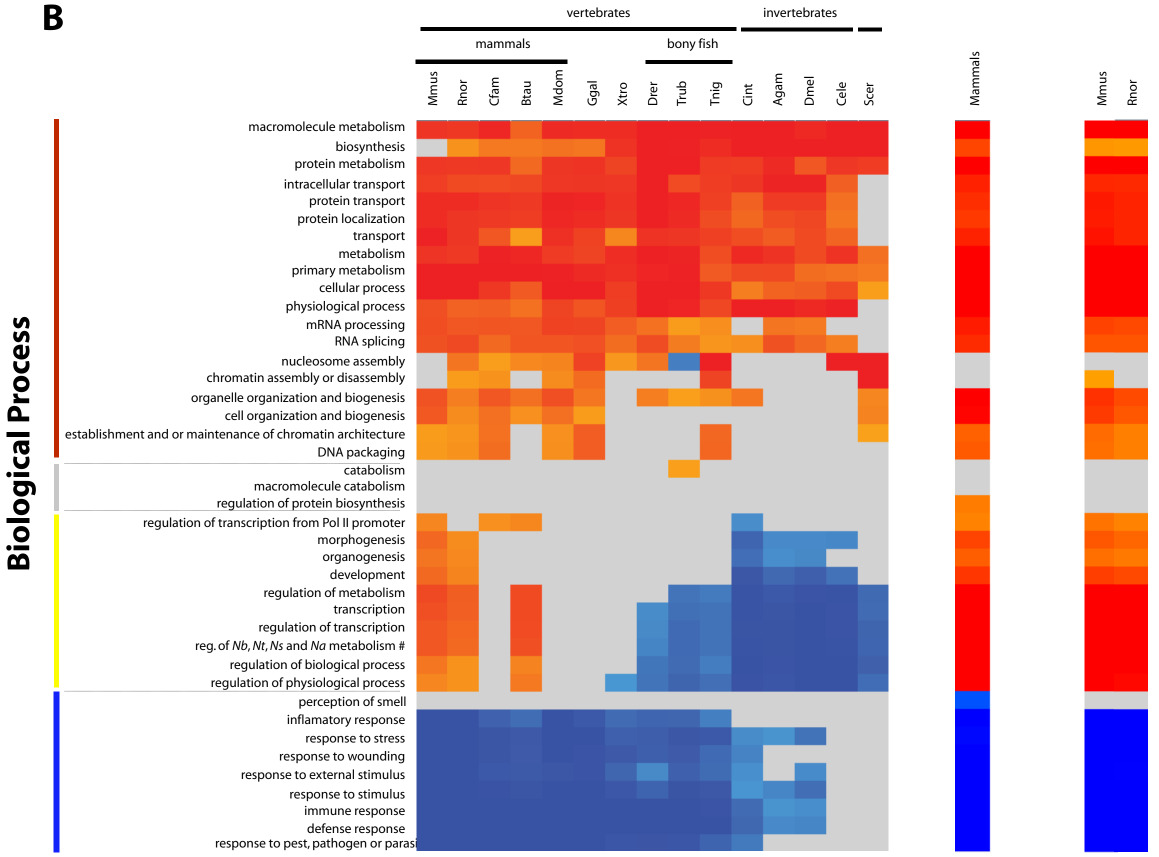

regulation of nucleobase, nucleoside, nucleotide and nucleic acid metabolism

Supplementary figure 6

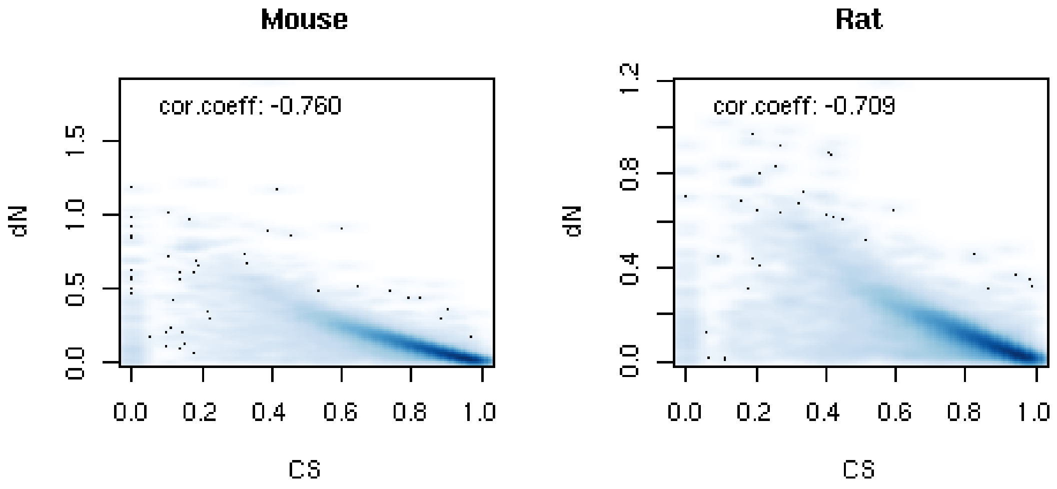

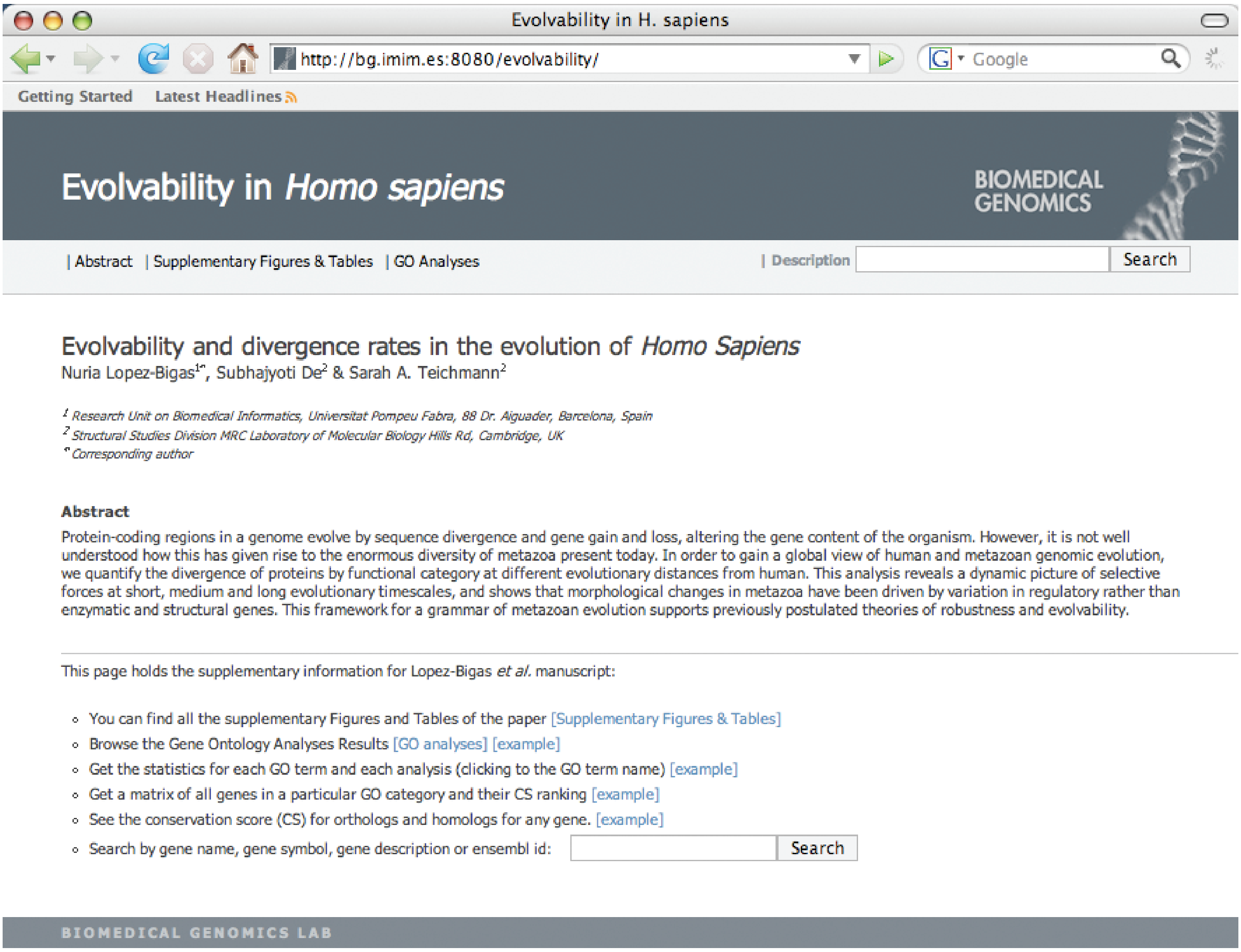

Evolvability in H. sapiens

http://bg.imim.es:8080/evolvability/

Getting Started Latest Headlines

Evolvability in *Homo sapiens*

BIOMEDICAL GENOMICS

| Abstract | Supplementary Figures & Tables | GO Analyses | Description [ ] Search

Evolvability and divergence rates in the evolution of *Homo Sapiens*

Nuria Lopez-Bigas[1*], Subhajyoti De[2] & Sarah A. Teichmann[2]

[1] *Research Unit on Biomedical Informatics, Universitat Pompeu Fabra, 88 Dr. Aiguader, Barcelona, Spain*
[2] *Structural Studies Division MRC Laboratory of Molecular Biology Hills Rd, Cambridge, UK*
[*] *Corresponding author*

Abstract

Protein-coding regions in a genome evolve by sequence divergence and gene gain and loss, altering the gene content of the organism. However, it is not well understood how this has given rise to the enormous diversity of metazoa present today. In order to gain a global view of human and metazoan genomic evolution, we quantify the divergence of proteins by functional category at different evolutionary distances from human. This analysis reveals a dynamic picture of selective forces at short, medium and long evolutionary timescales, and shows that morphological changes in metazoa have been driven by variation in regulatory rather than enzymatic and structural genes. This framework for a grammar of metazoan evolution supports previously postulated theories of robustness and evolvability.

This page holds the supplementary information for Lopez-Bigas *et al.* manuscript:

- You can find all the supplementary Figures and Tables of the paper [Supplementary Figures & Tables]
- Browse the Gene Ontology Analyses Results [GO analyses] [example]
- Get the statistics for each GO term and each analysis (clicking to the GO term name) [example]
- Get a matrix of all genes in a particular GO category and their CS ranking [example]
- See the conservation score (CS) for orthologs and homologs for any gene. [example]
- Search by gene name, gene symbol, gene description or ensembl id: [ ] Search

BIOMEDICAL GENOMICS LAB

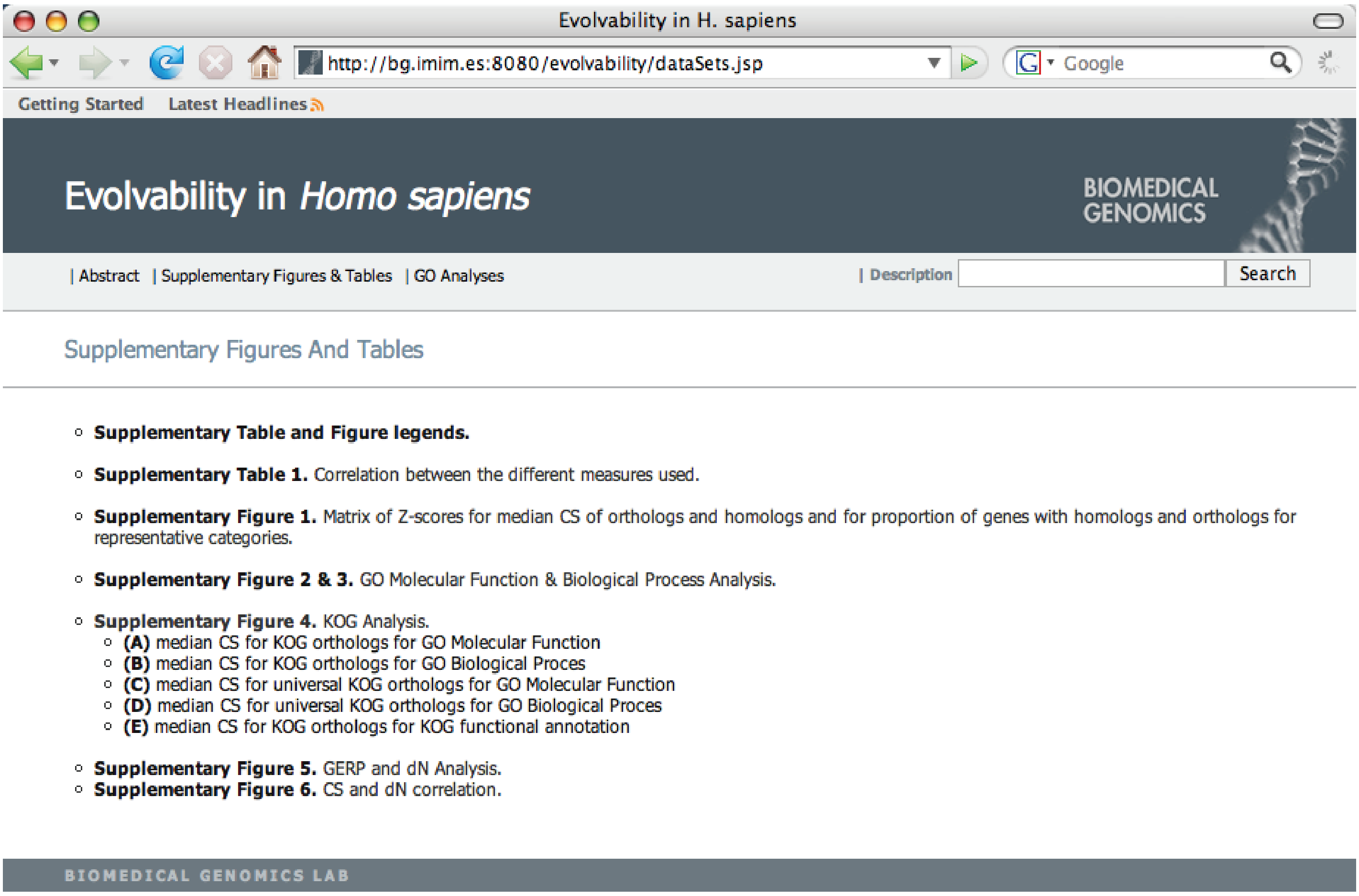

Evolvability in H. sapiens

http://bg.imim.es:8080/evolvability/dataSets.jsp

Getting Started Latest Headlines

Evolvability in *Homo sapiens*

BIOMEDICAL GENOMICS

| Abstract | Supplementary Figures & Tables | GO Analyses | Description Search

Supplementary Figures And Tables

- **Supplementary Table and Figure legends.**
- **Supplementary Table 1.** Correlation between the different measures used.
- **Supplementary Figure 1.** Matrix of Z-scores for median CS of orthologs and homologs and for proportion of genes with homologs and orthologs for representative categories.
- **Supplementary Figure 2 & 3.** GO Molecular Function & Biological Process Analysis.
- **Supplementary Figure 4.** KOG Analysis.
  - **(A)** median CS for KOG orthologs for GO Molecular Function
  - **(B)** median CS for KOG orthologs for GO Biological Proces
  - **(C)** median CS for universal KOG orthologs for GO Molecular Function
  - **(D)** median CS for universal KOG orthologs for GO Biological Proces
  - **(E)** median CS for KOG orthologs for KOG functional annotation
- **Supplementary Figure 5.** GERP and dN Analysis.
- **Supplementary Figure 6.** CS and dN correlation.

BIOMEDICAL GENOMICS LAB

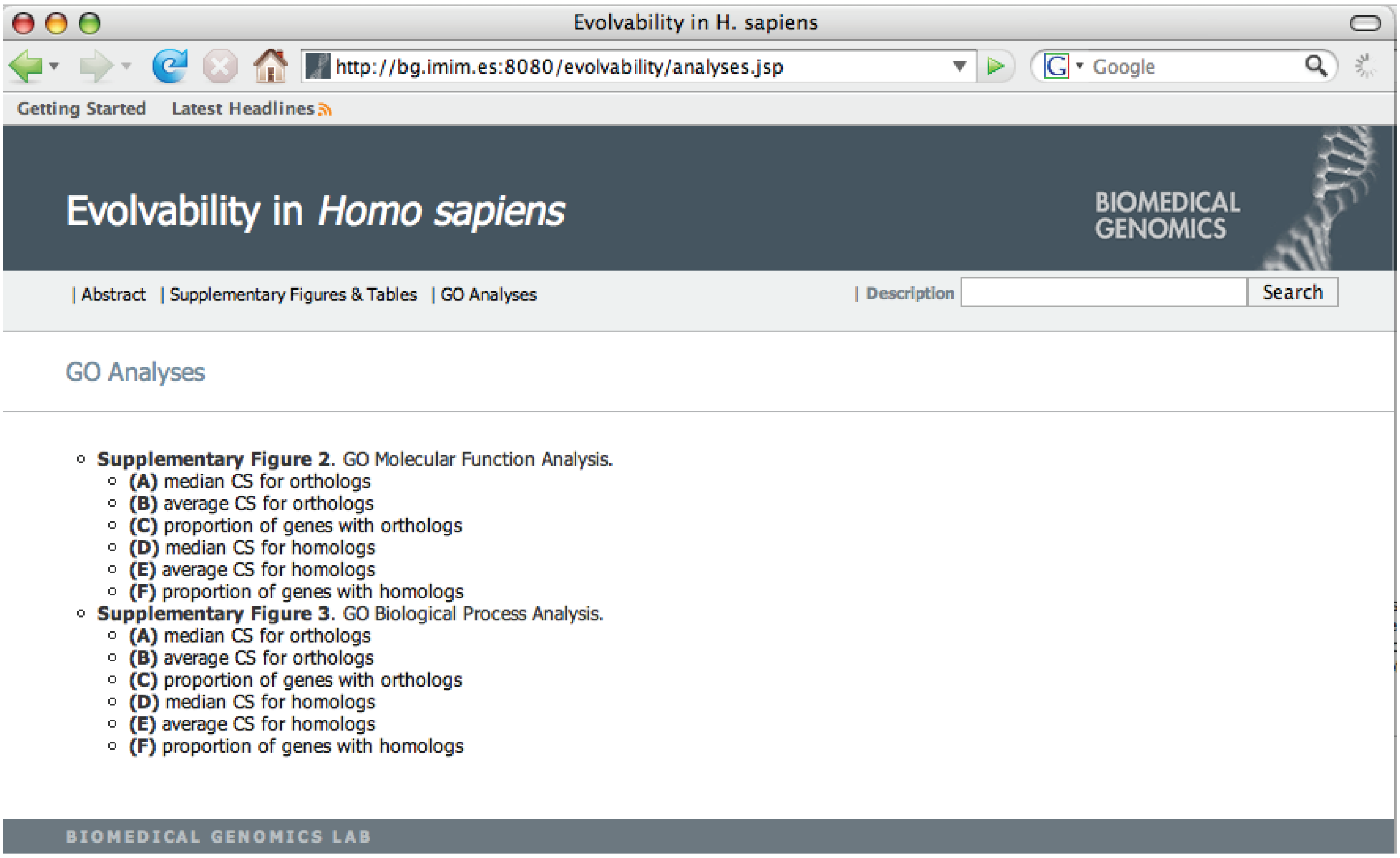

Evolvability in H. sapiens

http://bg.imim.es:8080/evolvability/analyses.jsp

Getting Started Latest Headlines

Evolvability in *Homo sapiens*

BIOMEDICAL GENOMICS

| Abstract | Supplementary Figures & Tables | GO Analyses | Description Search

GO Analyses

- **Supplementary Figure 2**. GO Molecular Function Analysis.
  - **(A)** median CS for orthologs
  - **(B)** average CS for orthologs
  - **(C)** proportion of genes with orthologs
  - **(D)** median CS for homologs
  - **(E)** average CS for homologs
  - **(F)** proportion of genes with homologs
- **Supplementary Figure 3**. GO Biological Process Analysis.
  - **(A)** median CS for orthologs
  - **(B)** average CS for orthologs
  - **(C)** proportion of genes with orthologs
  - **(D)** median CS for homologs
  - **(E)** average CS for homologs
  - **(F)** proportion of genes with homologs

BIOMEDICAL GENOMICS LAB

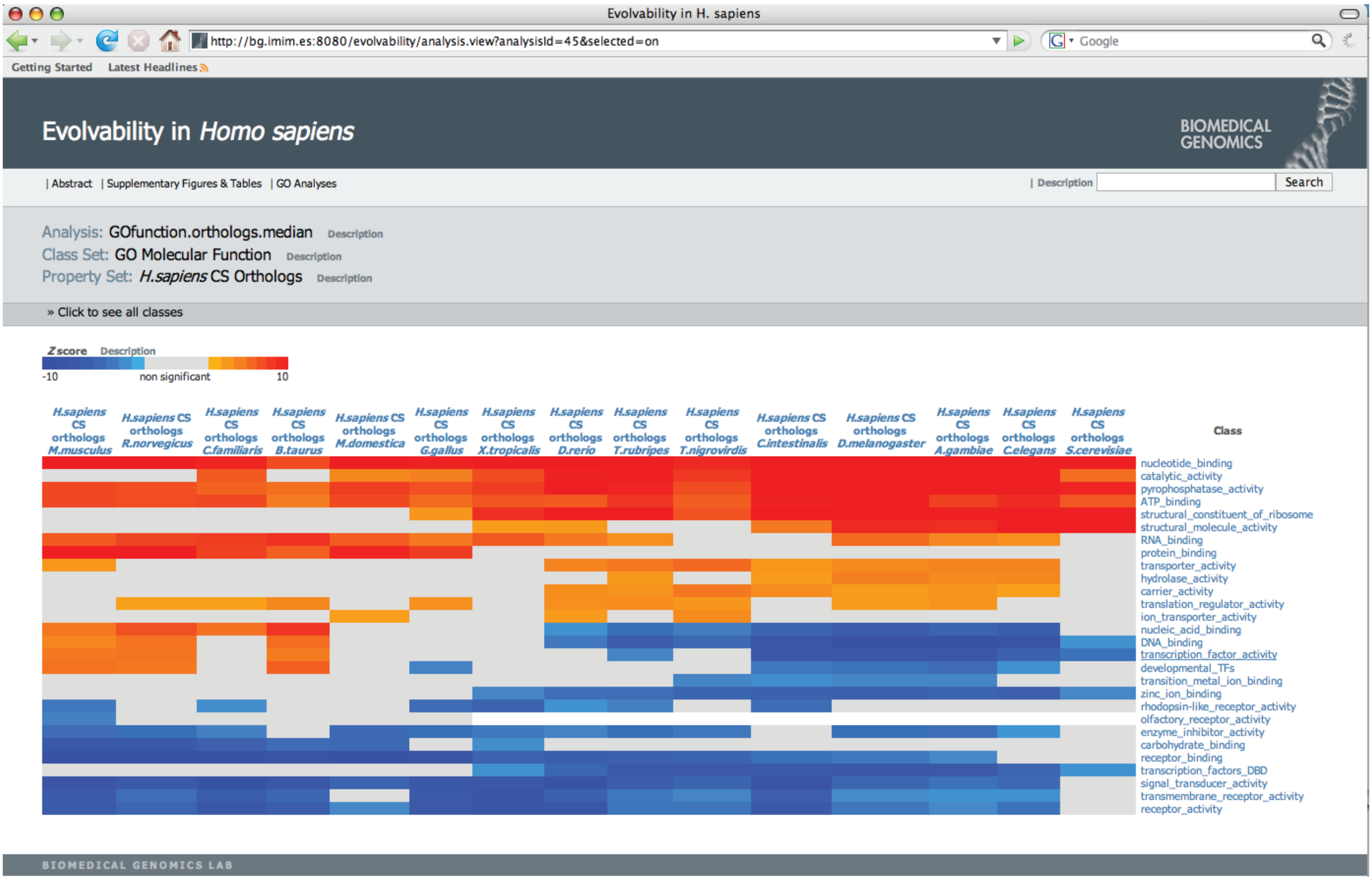

Description: *Z* Score

The *Z* score reveals how many units of the standard deviation a case is above or below the mean of the population. In this case it shows if the average or median CS of the genes in a particular GO category or the proportion of genes with homologs/orthologs differs significantly to the background population of genes.

Z scores are represented in a colour code where red represents high relative mean or median CS or high proportion of genes with homologs or orthologs for the category in a particular genome and blue represents low relative values. Gray means no statistically significant difference compared to the background of the whole genome. White cells denote that there is no ortholog/homolog with the GO term in the other organism.

Z score is defined as standardized distance between a statistics derived from a subset of elements from the population and a statistics bootstrapped from equally sized random samples taken from this population. It is expressed as:

$$Z_x = \frac{S - s_x}{\sigma_S}$$

where S is a statistics derived from a bootstrap, s_x is a statistics from a subset, and σ_S is a standard deviation from the bootstrap.

For continuous statistics σ is calculated as:

$$\sigma = \sqrt{\frac{\sum_{i=0}^{N} (X - x_i)^2}{N}}$$

where X is a mean of statistics derived from a bootstrap, x_i is a statistics from ith bootstrapping iteration and N is a number of elements in the population;

and for binomial ones as follows:

$$\sigma = \sqrt{\frac{p\ (1 - p)}{N}}$$

where p is a proportion in the population and N is a number of elements in the population.

Evolvability in H. sapiens

http://bg.imim.es:8080/evolvability/clazzProps.view?propertySetId

Getting Started Latest Headlines

Evolvability in *Homo sapiens*

BIOMEDICAL GENOMICS

| Abstract | Supplementary Figures & Tables | GO Analyses | Description Search

Property Set: *H.sapiens* CS Orthologs Description

Class: Catalytic_activity Description

Genes |

Z score Description

-10 non significant 10

Table description

Property	X	μ	σ	N	Z	p	
H.sapiens CS orthologs *M.musculus*	0.8642	0.8586	0.002	21,875	2.791	0.0053	
H.sapiens CS orthologs *R.norvegicus*	0.8494	0.8418	0.0022	21,875	3.3952	0.0007	
H.sapiens CS orthologs *C.familiaris*	0.8692	0.8546	0.0022	21,875	6.5008	< 1E-15	
H.sapiens CS orthologs *B.taurus*	0.8324	0.8232	0.0028	21,875	3.2169	0.0013	
H.sapiens CS orthologs *M.domestica*	0.7717	0.7578	0.003	21,875	4.6318	3.63E-06	
H.sapiens CS orthologs *G.gallus*	0.6993	0.6813	0.0036	21,875	4.964	6.91E-07	
H.sapiens CS orthologs *X.tropicalis*	0.6215	0.5956	0.0036	21,875	7.1285	< 1E-15	
H.sapiens CS orthologs *D.rerio*	0.5576	0.5272	0.0031	21,875	9.6644	< 1E-15	
H.sapiens CS orthologs *T.rubripes*	0.5728	0.5334	0.0035	21,875	11.1476	< 1E-15	
H.sapiens CS orthologs *T.nigrovirdis*	0.5069	0.4771	0.0037	21,875	8.0083	< 1E-15	
H.sapiens CS orthologs *C.intestinalis*	0.3698	0.3289	0.0035	21,875	11.8176	< 1E-15	
H.sapiens CS orthologs *D.melanogaster*	0.3571	0.3187	0.0035	21,875	10.9456	< 1E-15	
H.sapiens CS orthologs *A.gambiae*	0.3542	0.317	0.0036	21,875	10.1995	< 1E-15	
H.sapiens CS orthologs *C.elegans*	0.315	0.2706	0.0038	21,875	11.5793	< 1E-15	
H.sapiens CS orthologs *S.cerevisiae*	0.2412	0.2185	0.004	21,875	5.6728	1.41E-08	

BIOMEDICAL GENOMICS LAB

Description: *Z* Score Table

Column	Description
X	statistics value (mean, median or proportion) derived from sample
μ	mean of statistics values (derived from bootstrap or calculated from proportion)*
σ	standard deviation (derived from bootstrap or calculated from proportion)*
N	number of elements in the sample
Z	*Z* score value
p	*p* value
`*color*`	*Z* score's color code

*See: *Z* score description.

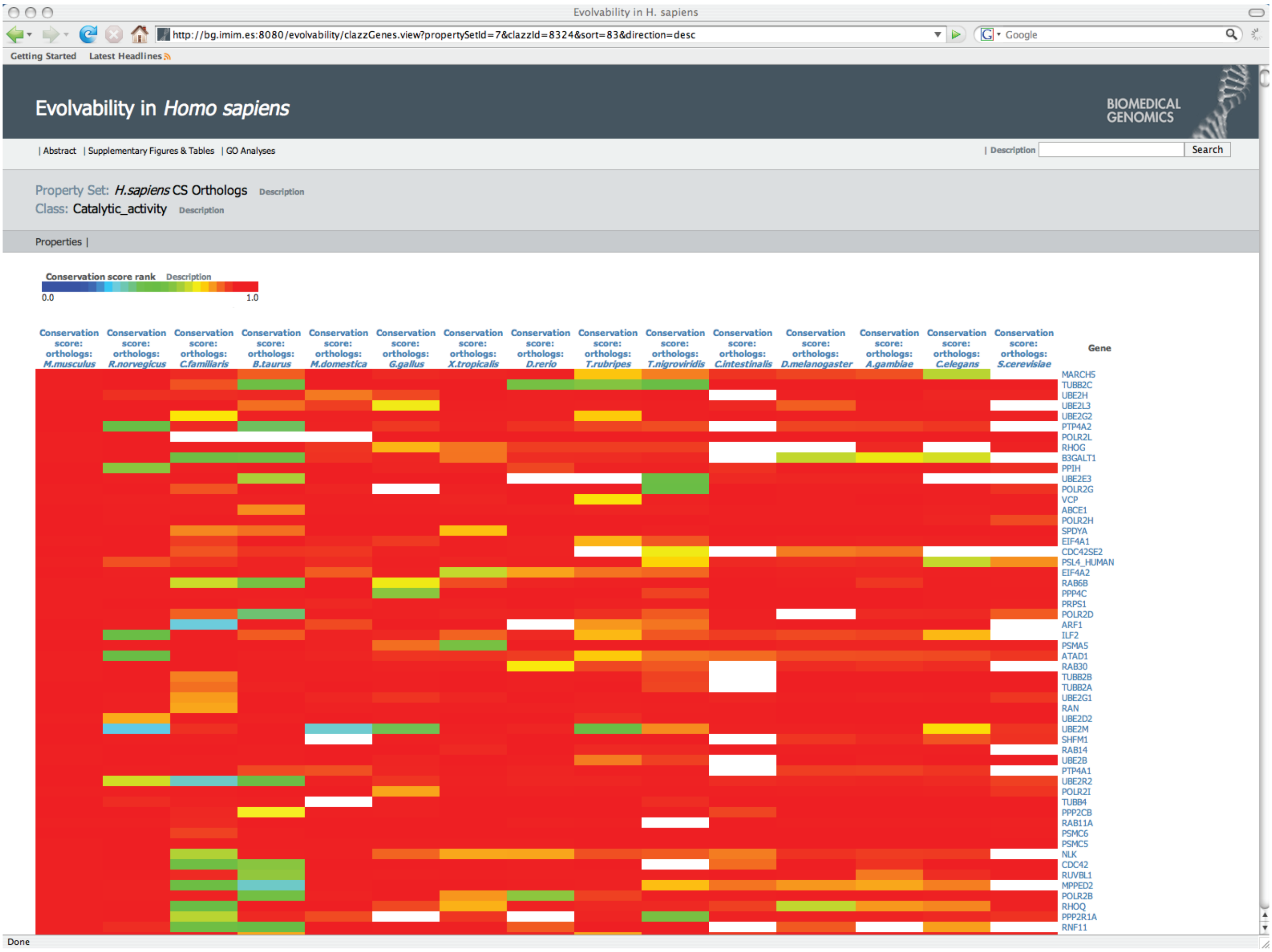

Description: Conservation Score Rank

All human genes with orthologs or homologs in the other genome are ranked according to their CS. The gene with the highest CS is shown in red and the one with the lowest CS in blue, with all others in intermediate colours according to their rank by CS. Thus, colours towards red mean high relative CS of the protein, green is medium relative CS and blue low relative CS. White cells denotes that this gene doesn't have an homolog or ortholog in that genome.

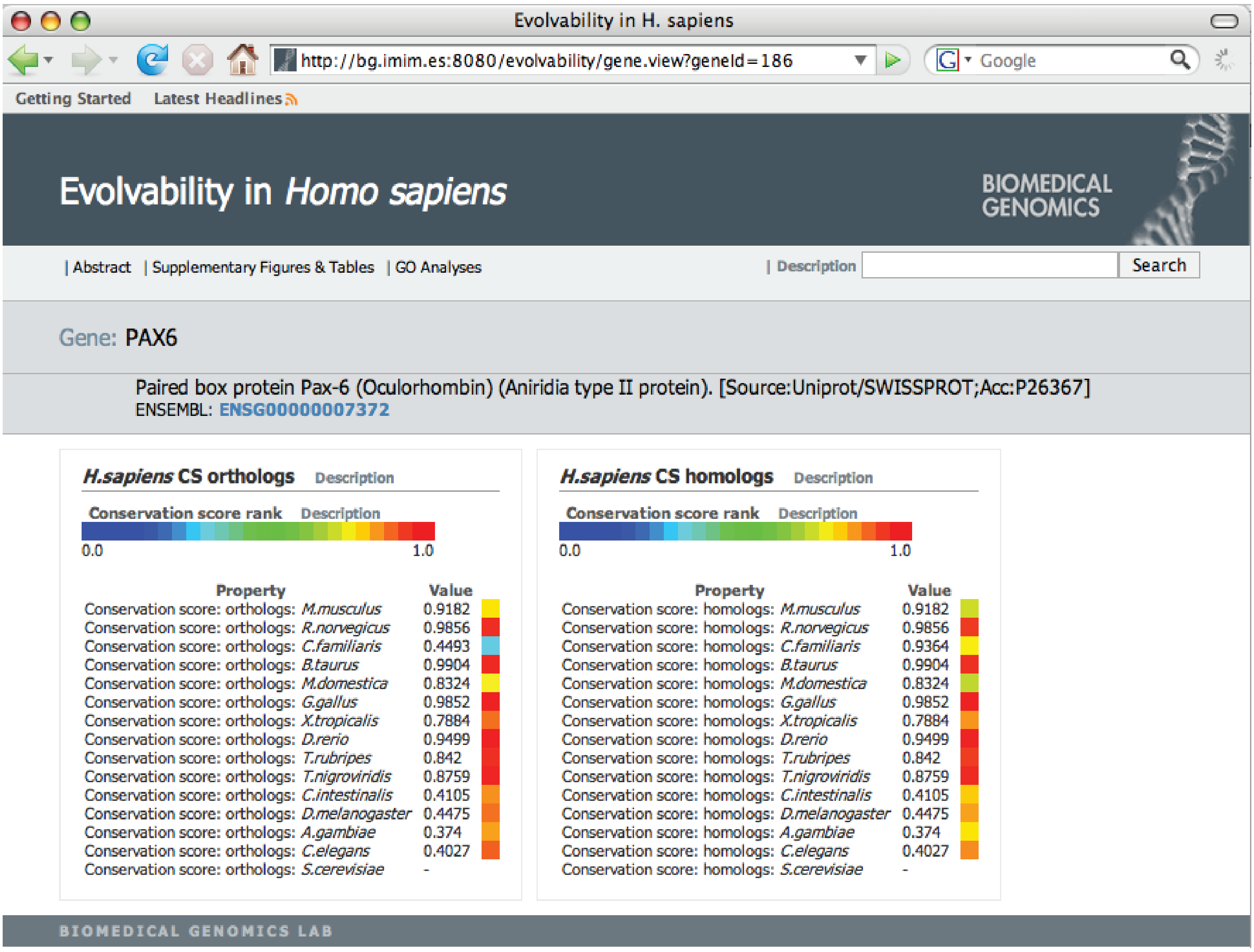

Evolvability in H. sapiens

http://bg.imim.es:8080/evolvability/gene.view?geneId=186

Getting Started Latest Headlines

Evolvability in *Homo sapiens*

BIOMEDICAL GENOMICS

| Abstract | Supplementary Figures & Tables | GO Analyses | Description [ ] Search

Gene: PAX6

Paired box protein Pax-6 (Oculorhombin) (Aniridia type II protein). [Source:Uniprot/SWISSPROT;Acc:P26367]
ENSEMBL: ENSG00000007372

***H.sapiens* CS orthologs** Description

Conservation score rank Description

0.0 1.0

Property	Value
Conservation score: orthologs: *M.musculus*	0.9182
Conservation score: orthologs: *R.norvegicus*	0.9856
Conservation score: orthologs: *C.familiaris*	0.4493
Conservation score: orthologs: *B.taurus*	0.9904
Conservation score: orthologs: *M.domestica*	0.8324
Conservation score: orthologs: *G.gallus*	0.9852
Conservation score: orthologs: *X.tropicalis*	0.7884
Conservation score: orthologs: *D.rerio*	0.9499
Conservation score: orthologs: *T.rubripes*	0.842
Conservation score: orthologs: *T.nigroviridis*	0.8759
Conservation score: orthologs: *C.intestinalis*	0.4105
Conservation score: orthologs: *D.melanogaster*	0.4475
Conservation score: orthologs: *A.gambiae*	0.374
Conservation score: orthologs: *C.elegans*	0.4027
Conservation score: orthologs: *S.cerevisiae*	-

***H.sapiens* CS homologs** Description

Conservation score rank Description

0.0 1.0

Property	Value
Conservation score: homologs: *M.musculus*	0.9182
Conservation score: homologs: *R.norvegicus*	0.9856
Conservation score: homologs: *C.familiaris*	0.9364
Conservation score: homologs: *B.taurus*	0.9904
Conservation score: homologs: *M.domestica*	0.8324
Conservation score: homologs: *G.gallus*	0.9852
Conservation score: homologs: *X.tropicalis*	0.7884
Conservation score: homologs: *D.rerio*	0.9499
Conservation score: homologs: *T.rubripes*	0.842
Conservation score: homologs: *T.nigroviridis*	0.8759
Conservation score: homologs: *C.intestinalis*	0.4105
Conservation score: homologs: *D.melanogaster*	0.4475
Conservation score: homologs: *A.gambiae*	0.374
Conservation score: homologs: *C.elegans*	0.4027
Conservation score: homologs: *S.cerevisiae*	-

BIOMEDICAL GENOMICS LAB

Description: Conservation Score Rank

All human genes with orthologs or homologs in the other genome are ranked according to their CS. The gene with the highest CS is shown in red and the one with the lowest CS in blue, with all others in intermediate colours according to their rank by CS. Thus, colours towards red mean high relative CS of the protein, green is medium relative CS and blue low relative CS. White cells denotes that this gene doesn't have an homolog or ortholog in that genome.
